# A Small Amount of Dietary Carbohydrate Can Promote the HFD-Induced Insulin Resistance to a Maximal Level

**DOI:** 10.1371/journal.pone.0100875

**Published:** 2014-07-23

**Authors:** Shuang Mei, Xuefeng Yang, Huailan Guo, Haihua Gu, Longying Zha, Junwei Cai, Xuefeng Li, Zhenqi Liu, Brian J. Bennett, Ling He, Wenhong Cao

**Affiliations:** 1 Nutrition Research Institute at Kannapolis, Department of Nutrition, Gillings School of Global Public Health, The University of North Carolina at Chapel Hill, Chapel Hill, North Carolina, United States of America; 2 Department of Nutrition, The University of North Carolina at Chapel Hill, Chapel Hill, North Carolina, United States of America; 3 Department of Nutrition and Food Hygiene, South Medical University, Guangzhou, China; 4 Department of Medicine, Taihe Hospital, Hubei University of Medicine, Shiyan, Hubei, China; 5 Department of Medicine, University of Virginia Health System, Charlottesville, Virginia, United States of America; 6 Department of Human Genetics, The University of North Carolina at Chapel Hill, Chapel Hill, North Carolina, United States of America; 7 Department of Pediatrics, Johns Hopkins University School of Medicine, Baltimore, Maryland, United States of America; 8 Department of Medicine (Endocrinology and Metabolism), Duke University School of Medicine, Durham, North Carolina, United States of America; Boston University School of Medicine, United States of America

## Abstract

Both dietary fat and carbohydrates (Carbs) may play important roles in the development of insulin resistance. The main goal of this study was to further define the roles for fat and dietary carbs in insulin resistance. C57BL/6 mice were fed normal chow diet (CD) or HFD containing 0.1–25.5% carbs for 5 weeks, followed by evaluations of calorie consumption, body weight and fat gains, insulin sensitivity, intratissue insulin signaling, ectopic fat, and oxidative stress in liver and skeletal muscle. The role of hepatic gluconeogenesis in the HFD-induced insulin resistance was determined in mice. The role of fat in insulin resistance was also examined in cultured cells. HFD with little carbs (0.1%) induced severe insulin resistance. Addition of 5% carbs to HFD dramatically elevated insulin resistance and 10% carbs in HFD was sufficient to induce a maximal level of insulin resistance. HFD with little carbs induced ectopic fat accumulation and oxidative stress in liver and skeletal muscle and addition of carbs to HFD dramatically enhanced ectopic fat and oxidative stress. HFD increased hepatic expression of key gluconeogenic genes and the increase was most dramatic by HFD with little carbs, and inhibition of hepatic gluconeogenesis prevented the HFD-induced insulin resistance. In cultured cells, development of insulin resistance induced by a pathological level of insulin was prevented in the absence of fat. Together, fat is essential for development of insulin resistance and dietary carb is not necessary for HFD-induced insulin resistance due to the presence of hepatic gluconeogenesis but a very small amount of it can promote HFD-induced insulin resistance to a maximal level.

## Introduction

Obesity is closely associated with insulin resistance, which is a necessary precursor and component of type 2 diabetes mellitus (T2DM). That means that insulin resistance is also an essential precursor of many major health problems associated with T2DM. These problems include cardiocerebrovascular disorders, non-alcoholic fatty liver, dementia, asthma, some cancers, and aging [1]–[9]. In order to prevent all these major health problems, it is imperative to fully understand how insulin resistance is caused by different dietary components.

High fat diet (HFD, >37% fat in the diet) is associated with insulin resistance in humans [10] and can consistently induce obesity and insulin resistance in animal models [11]–[15]. Therefore, fat has been generally blamed for the pandemic development of T2DM. However, some studies have shown that fat-rich diets can actually protect against insulin resistance and T2DM [16] and the fat-rich Atkins diet and other diets are very popular among many people. That means the jury is still out there about the exact role of dietary fat in the development of insulin resistance and T2DM. Similarly, the role of dietary carbohydrates (carbs) has not been fully established or defined. Some studies have shown that carb-rich diets promote development of insulin resistance and T2DM [17],[18] while others have shown that high-carb diet is protective against insulin resistance and T2DM compared to HFD [19], and low-carb diet is not necessarily protective against insulin resistance and diabetes [20],[21]. These conflicting results demonstrate the complexity and difficulty in defining the role of each dietary component in insulin resistance and T2DM. In order to accurately address the roles of dietary fat and carbs in insulin resistance and T2DM, several key questions must be answered. First, are dietary carbs necessary for the HFD-induced insulin resistance? Second, how much carb is too much or sufficient to promote the HFD-induced insulin resistance? Third, is fat essential for insulin resistance? In this study, we addressed these questions and the associated mechanisms.

## Materials And Methods

Antibodies against phosphorylated Akt, total Akt, or β-actin were from Sigma (St. Louis, MO). Antibodies against IRS-1^Ser636^, UCP-1 were from Abcam (Cambridge, MA). Mouse IgG, and rabbit IgG were obtained from Santa Cruz Biotechnology Inc. (Santa Cruz, CA). RNeasy Mini Kit was from Qiagen (Valencia, CA). GSH/GSSG ratio measuring kit was from OxisReaserch (UK). MDA and MnSOD activity assay kits were from Cell Biolabs (USA). Other materials were all obtained commercially and are of analytical quality.

### Animal Experiments

C57BL/6 (B6) mice (male, 6–9 week old) were fed the standard Prolab RMH 3000 chow diet (CD, 65.1% calories from carb, 23.1% calories from proteins, and 11.8% calories from fat) *ad libitum* or high fat diet (HFD, 58%) plus 0.1%, 5%, 10%, or 25.5% carbohydrates (Carbs, maltodextrin) for 5 weeks and kept at 12 h light and 12 h dark cycles as described [13]. The total calorie densities of HFD with different amount of carb were the same by adjusting the amount of proteins (casein) in the diets. All HFD were from Research Diets (Cat#: D11101101, D11101102, D11101103, and D12331). After an 8 h-fast at the end of every week, blood glucose, body weight, food intake and water intake were measured weekly. Some mice on HFD + 0.1% carb were treated with adenoviral shRNA against p300 (10^9^ pfu/mouse) or the control shRNA at the beginning of week 5 as detailed previously [22]. After an 8 h-fasting at the end of the 5^th^ week, insulin tolerance test (ITT) were performed by injecting human insulin (0.75 U/kg body weight), followed by measurements of blood glucose (0 min, 30 min, 60 min, 90 min, and 120 min) with a glucosemeter. Blood samples were also collected. Sera were obtained after centrifugation at 3000×g for 10 min and stored at −80°C. Serum triglyceride (TG) and free fatty acids (FFA) were measured with kits from Cayman Chemical (Cat No: 10010303 and 700310). Serum cholesterols were measured with Amplex red cholesterol assay kit. Serum endogenous insulin was detected with ELISA kits from Millipore (Cat No: EZRMI-13K and EZRMGH-45K). Assays were performed according to the manuals from the manufacturers. Tissues from liver and gastrocnemius were obtained after animals were dead and stored at −80°C. All animal studies were approved by the institutional animal care and use committee of The University of North Carolina at Chapel Hill and fully complied with the guidance from the National Institutes of Health.

### Measurement Of Food Consumption

Food consumption was measured by subtracting the amount of the food left and the initial amount of the food supplied. Energy intakes were calculated on the basis of 3.81 kcal/g for the CD (Lab Diet, Richm*on*d, IN, USA) and 5.56 kcal/g for HFD groups. Calories/BW (g)/day in chow diet group  =  (food supplied-food left)*3.81/body weight/7 days. Calories/BW (g)/day in HFD groups  =  (food supplied-food left)*5.56/body weight/7 days.

### Measurements Of Lipids In Tissues And Plasma Insulin

Triglyceride contents (TG) in liver and muscle were measured with kits from Cayman Chemical (Cat No: 10010303) [14]. Endogenous plasma insulin level was quantified with an ELISA kit from Millipore (Cat No: EZRMI-13K and EZRMGH-45K). Assays were performed according to manuals from the manufacturers.

### Immunoblotting

Thirty micrograms of total tissue proteins were denatured at 95°C for 5 min in loading buffer (60 mM Tris, 2.5% SDS, 10% glycerol, 5% beta-mercaptoethanol, 0.01% bromphenol-blue) and subjected to 10% SDS-PAGE. Proteins were transferred to PVDF membranes and blocked with TBS buffer containing 0.05% Tween 20 (TBS-T) and 5% nonfat milk for 1 h. After washing in TBS-T, membranes were probed with specific 1st antibodies against target proteins (rabbit) or β-actin (mouse) (1∶1000) overnight at 4°C. Membranes were then washed with TBS-T and incubated with polyclonal secondary goat anti-rabbit or rabbit anti-mouse antibodies (1∶5000; Santa Cruz Biotechnology) for 1 h at room temperature. After three washes with TBS-T, membranes were treated with ECF substrates, according to the manufacturer's protocol (Thermo Scientific). Fluorescent bands were visualized and then quantified by densitometry analysis using ImageQuant version 5.2 software from GE Healthcare.

### Rna Extraction And Real-Time Pcr

Total RNAs were extracted from tissues with an RNeasy Mini Kit (Qiagen), and reverse-transcribed into cDNAs, which were quantified by TaqMan Real-time PCR with probes and primers were Srebp1(NM_011480.3): GGTTTTGAACGAC ATCGAAGA (forward) and CGGGAAGTCAC TGTCTT GGT (reverse). Srebp2/NM_033218.1: ACCTAGACCTCGCCAAAG GT (forward) and GCACGGATAAGCAGGTT TGT (reverse). Fas/NM_007988.3: CCAAATCCAACATGGGACA (forward) and TGCTCCAGGGATAACAGCA (reverse). Results were normalized to levels of M36B4.

### Measurement Of Peroxidized Lipids

Levels of peroxidized lipids in liver and muscle were indirectly determined by measuring levels of malondialdehyde (MDA), a byproduct of lipid peroxidation with a commercialized kit (Northwest Life Science Specialties, USA) [23]. The level of MDA was determined by quantifying the absorbance at 532 nm and presented as µM/mg proteins.

### Measurement Of Mnsod Activity

MnSOD activity in liver and muscle was determined by using a commercialized kit (Cayman Chemical Company) following the manufacturer's instructions. Briefly, xanthine oxidase and hypoxanthine were used to generate superoxide radicals that were then detected by tetrazolium salt and quantified at 540 nm with a microplate analyzer (Cell Biolabs, USA). One unit of SOD was defined as the amount of enzyme required to inhibit the distmutation of the superoxide radical by 50%.

### Measurement Of Glutathione (gsh)/glutathione Disulfide (gssg) Ratio

Levels of GSH and GSSG in tissue lysates were determined with a kit from OXIS International, Inc. (Foster City, CA) and normalized to protein levels [24]. The GSH/GSSG ratio was calculated according to the instruction provided by the manufacturer. In brief, measurements of GSH and GSSG and calculation of the GSH/GSSG ratio were performed in the following four steps: 1) determination of reaction rate by time; 2) construction of calibration curves; 3) calculation of the analyst concentrations; and 4) calculation of the GSH/GSSG ratio: GSH/GSSG ratio  =  (GSH- 2GSSG)/GSSG).

### Statistical Analysis

Data were presented as mean ± SD compared among different groups by one way ANOVA with Scheffe's post-hoc test. (San Diego, CA). Differences at values of p<0.05 were considered significant.

## Results

### Dietary Carbohydrates (carbs) Are Not Necessary For The Hfd-Mediated Body Weight Gain But Can Promote Body Weight Gain In A Dose-Dependent Manner In Mice On Hfd

**Figure 1:**
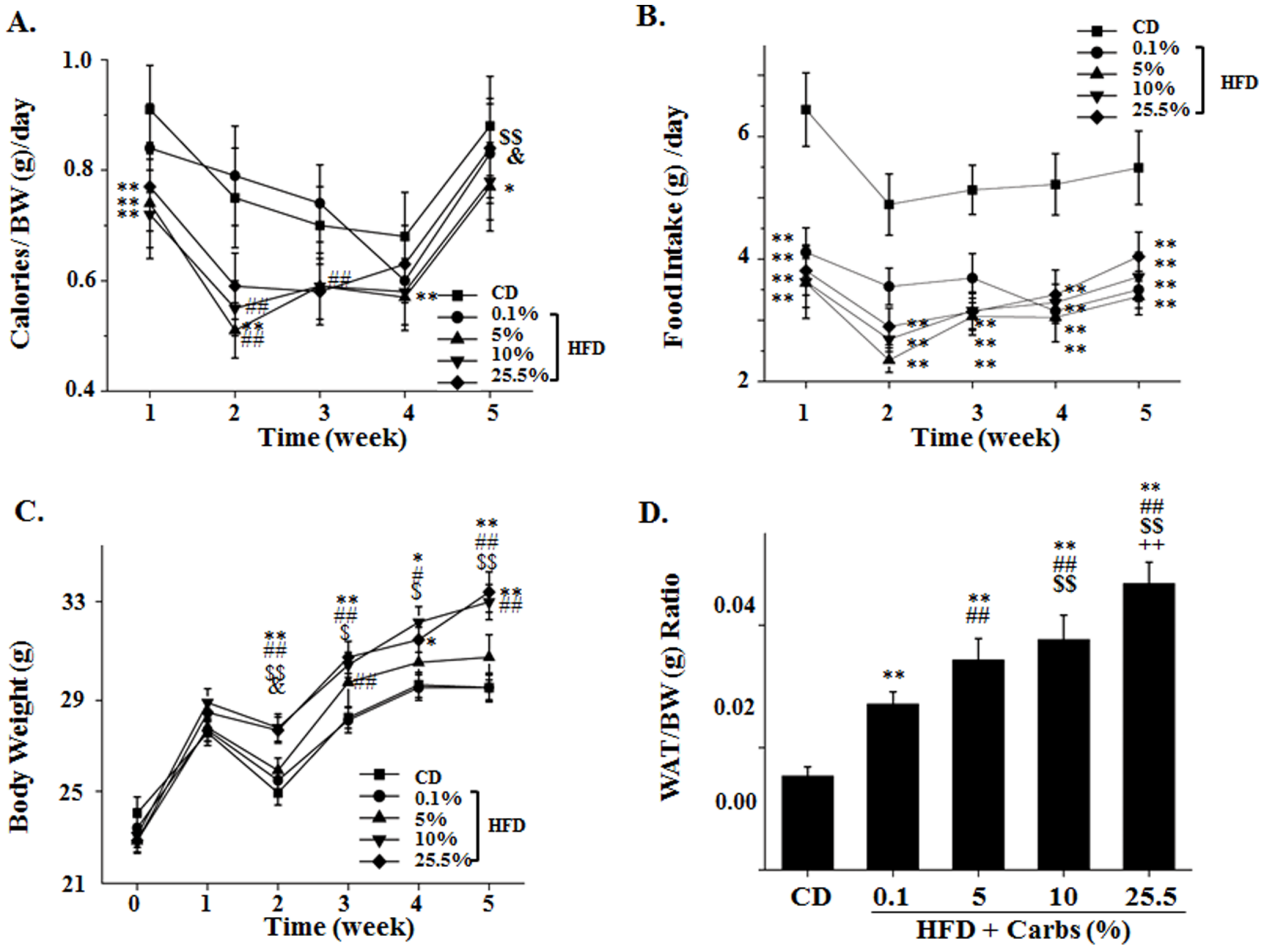
Dietary carbohydrates (carbs) are not necessary for the high fat diet (HFD)-mediated body weight gain but can promote the HFD-mediated weight gain in a dose-dependent manner. C57BL/6 (B6) mice were fed the regular chow diet (CD) *ad libitum* or HFD plus 0.1%, 5%, 10% or 25.5% carbs for 5 weeks. (A–B) Food and calorie intakes were recorded weekly. The ratio of food calories over body weight daily was calculated. *: P<0.05 CD vs. either HFD +5% carbs or HFD +10% carbs. **: P<0.01 vs. CD. ##: P<0.01 vs. HFD +0.1% carbs. $$: P<0.01 HFD +5% carbs vs. HFD +0.1% or HFD +25.5% carbs. &: P<0.05 HFD +10% carbs vs. HFD +25.5% carbs. (C) Body weight was measured weekly. *: P<0.05 vs. CD. **: P<0.01 vs. CD. #: P<0.05 vs. HFD +0.1% carbs. ##: P<0.01 vs. HFD +0.1% carbs. $: P<0.05 HFD +5% carbs vs. HFD +25.5% carbs. $$: P<0.01 HFD +5% carbs vs. HFD +25.5% carbs. &: P<0.05 HFD +10% carbs vs. HFD +25.5% carbs. (D) Epididymis fat pads were collected and weighed. The ratio of epididymis fat over body weight was calculated. Results represent mean ± SD of 10 mice/group. **: P<0.01 vs. CD. ##: P<0.01 vs. HFD +0.1% carbs. $$: P<0.01 vs. HFD +5% carbs. ++: P<0.01 vs. HFD +10% carbs.

It is established that high sucrose- high fat-diet can cause body weight gain (obesity) while the regular chow diet that contains 65.1% calories from carbs does not [11],[12]. However, it has not been defined whether or not dietary carbs are necessary for HFD induction of body weight gain and how much dietary carb is sufficient to promote body weight gain in animals on HFD. The answer for this question is important because many people are currently practicing or promoting low carb diets or high fat (Atkins) diets with mixed results [25]–[28]. To answer this question, we fed B6 mice with the regular chow diet, HFD (58% fat) + 0.1% carbs, HFD (58% fat) + 5% carbs, HFD (58% fat) + 10% carbs, or HFD (58% fat) plus 25.5% carbs for 5 weeks. Different amounts of proteins were added to achieve equal calories per gram of HFD. Calorie intake and body weight were evaluated once a week. As shown in **Fig. 1A–B**, mice on all HFD groups took less food and fewer calories than the mice on chow diet. At the end of 5 weeks, the decrease of calorie intake in mice on HFD was proportional to the amount of dietary carbs. But, animals on HFD gained more weight when the level of dietary carbs was increased (**Fig. 1C**). Specifically, animals with HFD + 0.1% carbs did not have an absolute gain in body weight compared to those on chow diet, but did gain more body weight per unit of calorie intake considering their lower calorie intake. Mice on HFD with 5% carbs caused absolutely more body weight gain and the HFD with 10% carbs caused equal amount of body weight gain as the HFD with 25.5% carbs, which is the standard level in experimental Surwit HFD in animals [11],[12]. It should be noted that mice on CD and all HFD groups had or tended to have reduced food intake and body weight in week 2 for an unknown reason. To determine the body composition, we measured the ratio between white fat (epididymis fat) over body weight. As shown in **Fig. 1D**, the white fat proportion was increased in mice on HFD with little (0.1%) dietary carbs, but the increase was further enhanced by dietary carbs in a dose-dependent manner. Serum levels of cholesterol, triglyceride (TG), and free fatty acids (FFA) were increased in all groups of HFD (data not shown) as usual. Together, these results show that dietary carbs are not essential for the HFD-induced body fat gain but can enhance the HFD-induced body fat gain in a dose-dependent manner, and a very small amount of carbs (10%) in the HFD can cause the maximal level of body weight gain.

### Dietary Carbs Are Not Necessary For Hfd To Induce Insulin Resistance But Can Aggravate Insulin Resistance In A Dose-Dependent Manner

**Figure 2:**
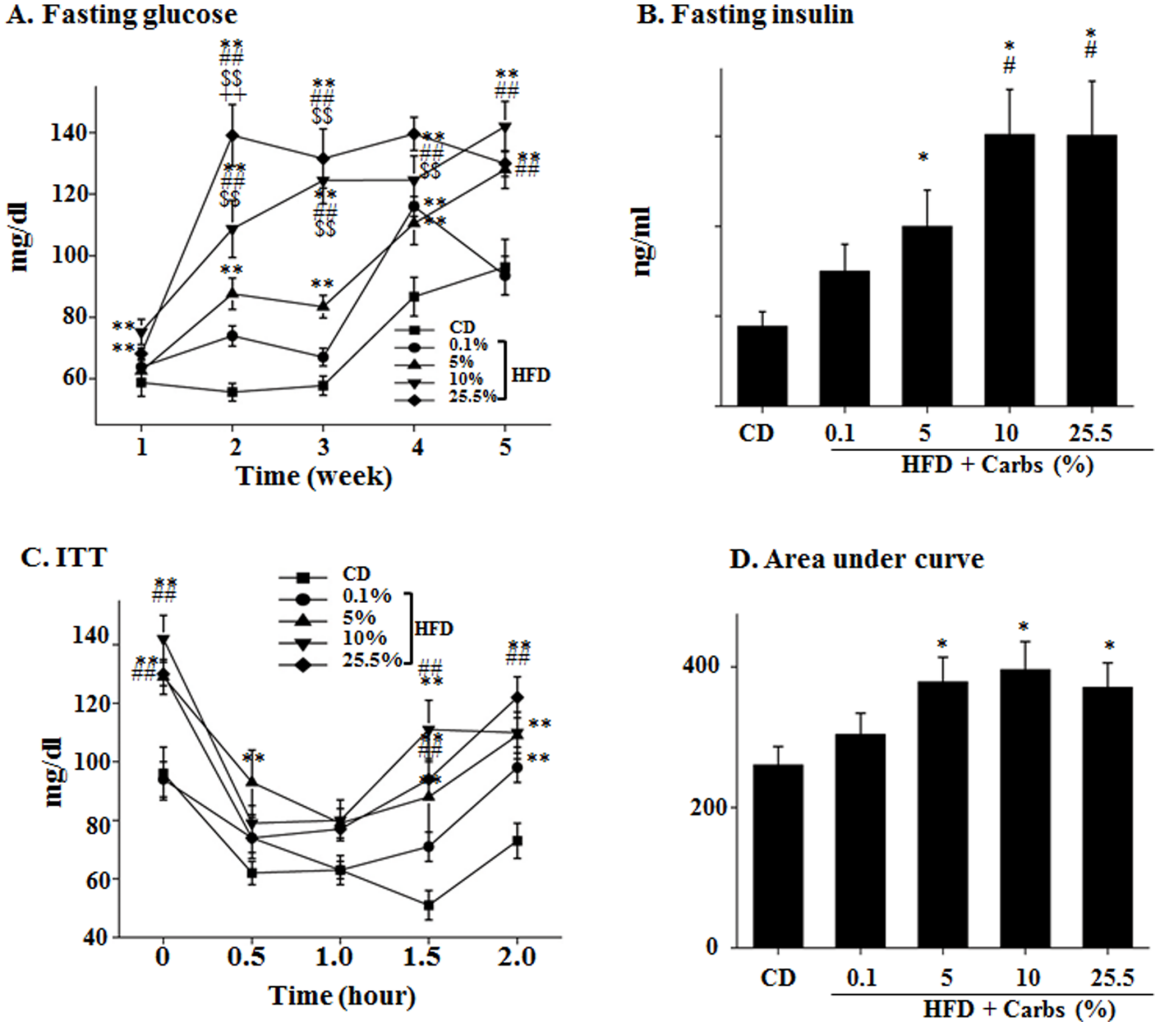
Dietary carb is not necessary for the HFD-induced insulin resistance but a small amount of it can induce a maximal level of insulin resistance in mice on HFD. (**A**) Fasting blood glucose was measured once a week after an 8-hour fast. (**B**) Plasma level of insulin was evaluated at the end of the 5-week experiment after an 8 hour fast. (**C–D**) Insulin tolerance test (ITT) was performed at the end of the 5-week experiment after an 8 hour fast and the area under curve was calculated. Results represent mean ± SD of 8 mice/group. *: P<0.05 vs. CD. **: P<0.01 vs. CD. #: P<0.05 vs. HFD +0.1% carbs. ##: P<0.01 vs. HFD +0.1% carbs. $$: P<0.01 vs. HFD +5% carbs. ++: P<0.01 vs. HFD +10% carbs.

It has been shown that high fat and high carbohydrate diet can consistently induce insulin resistance although its effect on induction of obesity and diabetes may vary a lot in B6 mice [11],[29]. To determine the role of dietary carbs in the HFD-induced insulin resistance, we evaluated insulin sensitivity in the animals described above by measuring fasting levels of blood glucose and insulin and performing ITT in B6 mice. As shown in **Fig. 2A**, fasting blood glucose level was increased in mice of all HFD groups including the HFD with little (0.1%) carbs starting from week 1, and the increase was promoted by dietary carbs in a dose-dependent manner. Similarly, fasting plasma insulin level was also increased in mice of all HFD groups, and the increase reached a maximal level when the carbs in the HFD reached 10% (**Fig. 2B**). As shown in **Fig. 2C–D**, insulin tolerance was significantly decreased in mice on HFD with little (0.1%) dietary carbs, and addition of carbs to HFD worsened insulin tolerance in a dose-dependent manner. Together, these results show that dietary carbs are not essential for the HFD-induced insulin resistance but can promote the HFD-induced insulin resistance dramatically, and 10% calories from dietary carbs can promote insulin resistance to a maximal level in mice on HFD.

### Hfd Causes Insulin Resistance In Liver And Skeletal Muscle With Or Without Dietary Carb

**Figure 3:**
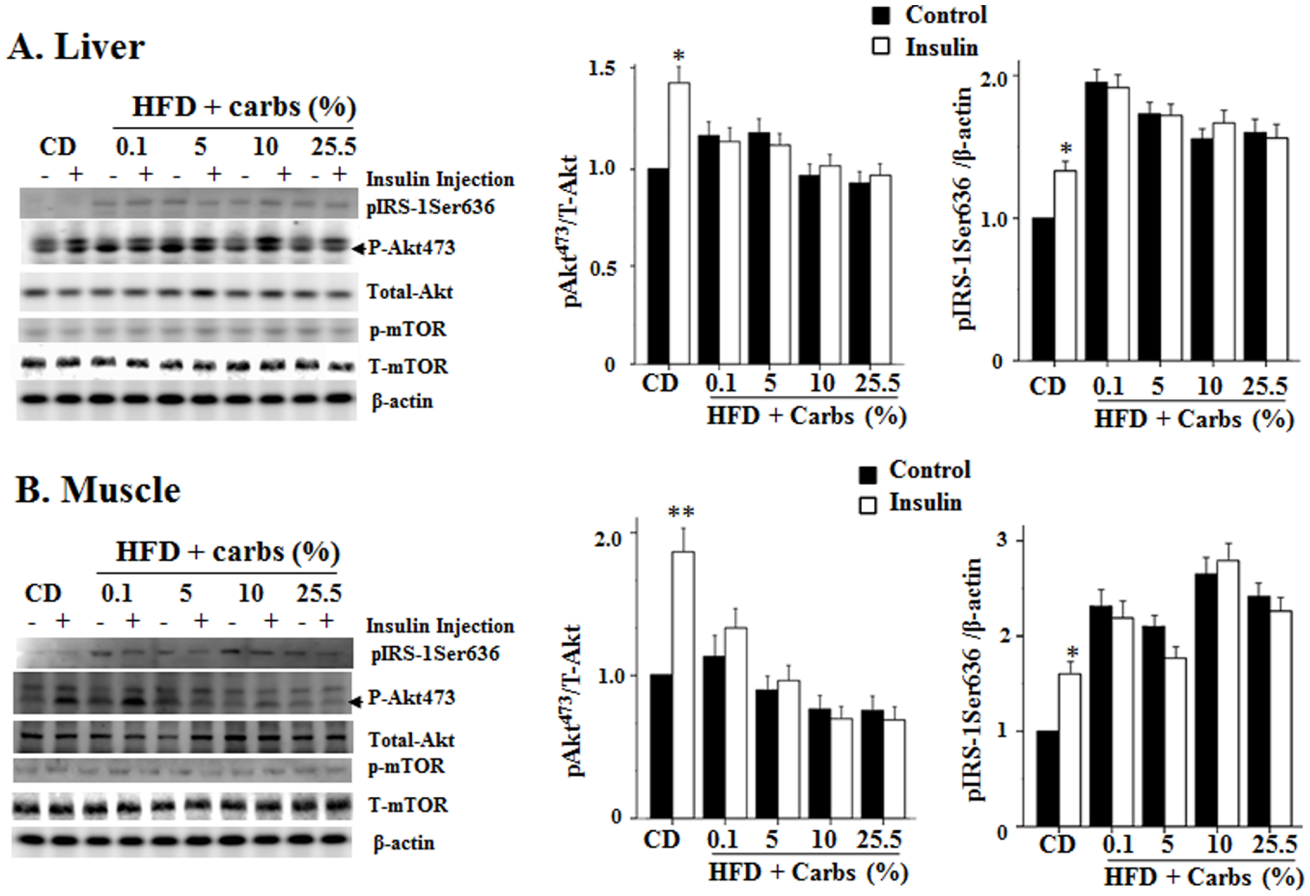
Dietary carb is not necessary for the HFD-induced insulin resistance. At the end of the 5-week experiment, animals were fasted for 8 h. Liver (**A**) and skeletal muscle samples (**B**) were collected promptly after animals were sacrificed. Levels of total and phosphorylated IRS1, total and phosphorylated Akt, total and phosphorylated mTOR, and β-actin were detected with immunoblotting. The level of each target protein was quantified and normalized to control. The average of 3 mice was set at 1. Results were presented as mean ± SD of 6 animals/group for Akt and 3 animals for IRS1. *: P<0.05 vs. no acute insulin treatment in control. **: P<0.01 vs. no acute insulin treatment in control.

To determine whether dietary carbs are necessary for HFD to induce insulin resistance in metabolically active tissues, we examined the levels of serine phosphorylation of IRS1 and phosphorylation of Akt in liver and gastrocnemius. As shown in **Fig. 3**, HFD elevated serine phosphorylation of IRS1 in both liver and gastrocnemius and the elevation was not obviously influenced by the amount of dietary carbs in HFD. Similarly, Akt phosphorylation induced by acute insulin challenge was blunted by HFD with or without dietary carbs (**Fig. 3**). It is noteworthy that mTOR activation was not influenced by the diets (**Fig. 3A–B**). Together, these results indicate that HFD can induce insulin resistance in metabolically active tissues with or without dietary carbs and evaluation of intra-tissue insulin signaling components can only tell whether or not insulin resistance is present but cannot tell the exact extent of insulin resistance like ITT.

### Fatty Acid/fat Is Necessary For The Development Of Insulin Resistance Induced By The Chronic Exposure To A Pathological Level Of Insulin

**Figure 4:**
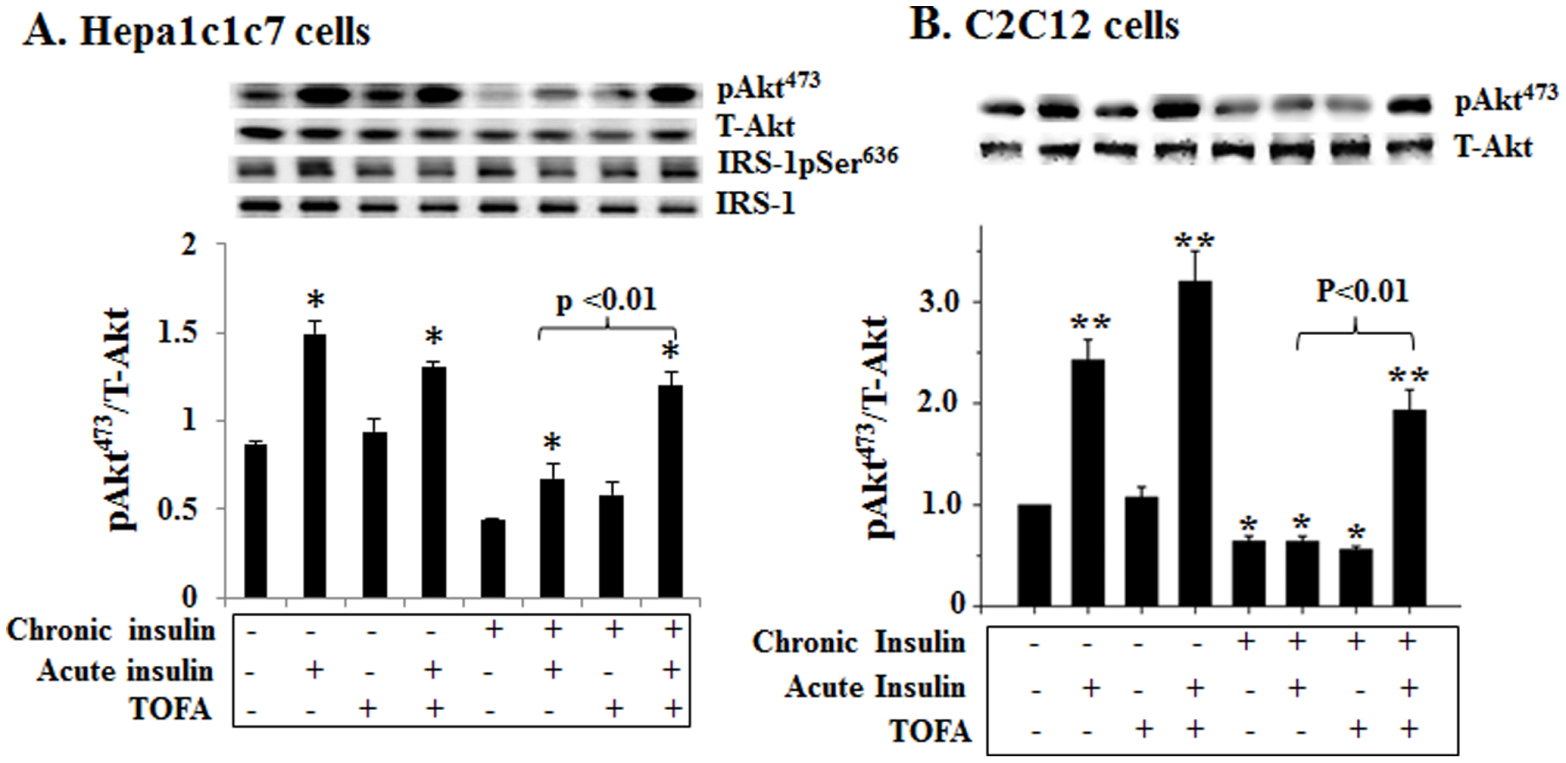
Fatty acid is essential for the development of insulin resistance induced by chronic exposure to insulin. Hep1c1c7 (**A**) and differentiated C1C12 (**B**) cells were pretreated with TOFA (30.8 µM) for 50 min in media containing 25 mM glucose before the incubation with insulin (1 nM, chronic insulin treatment) as noted in the continuous presence or absence of TOFA for another 12 h. After an extensive washing with warm PBS, cells were exposed to insulin (5 nM) for 15 min (acute insulin treatment) as noted, followed by evaluations of phosphorylated and total target proteins by using immunoblotting and quantification. No sera were added to the media throughout the whole experiment. Results represent mean ± SE of 3 independent experiments. *: P<0.05 vs. no acute insulin treatment. **: P<0.01 vs. no acute insulin treatment.

As described above, all animals on HFD with or without dietary carbohydrates developed insulin resistance while the animals on chow diet that is a typical high carb (65.1%) and low fat diet (11.8%) did not have insulin resistance. Surwit et al has previously shown that mice on sucrose diet without fat does not intake excess calories and their plasma levels of glucose and insulin are not affected [30]. We and others have previously shown that insulin plays an essential role in the HFD-induced insulin resistance [13],[14],[31]. We asked how important fat was in the development of insulin resistance induced by the chronic exposure to a pathological level of insulin (hyperinsulinemia) by depriving cells of exogenous (no sera was added) and endogenous fatty acids (by inhibition of fatty acid synthesis). As shown in **Fig. 4A**, in the absence of chronic exposure to insulin, the acute insulin treatment induced robust Akt phosphorylation in the presence or absence of fatty acid synthesis inhibitor TOFA in hepatocytes. Note: the effect of TOFA on fat synthesis should be minimal in such a short time (15 min) as predicted. In contrast, in the presence of chronic exposure to a pathological level of insulin, the acute insulin treatment induced moderate Akt phosphorylation in the absence of TOFA but stimulated robust Akt phosphorylation in the presence of TOFA (**Fig. 4A**). TOFA alone did not influence Akt phosphorylation (**Fig. 4A**). Similar results were observed in cultured myocytes (**Fig. 4B**). Together, these results demonstrate that fatty acid/fat is essential for the development of insulin resistance induced by the chronic exposure to a pathological level of insulin.

### Hfd With Or Without Dietary Carbs For 5 Weeks Induces Ectopic Fat Accumulation

**Figure 5:**
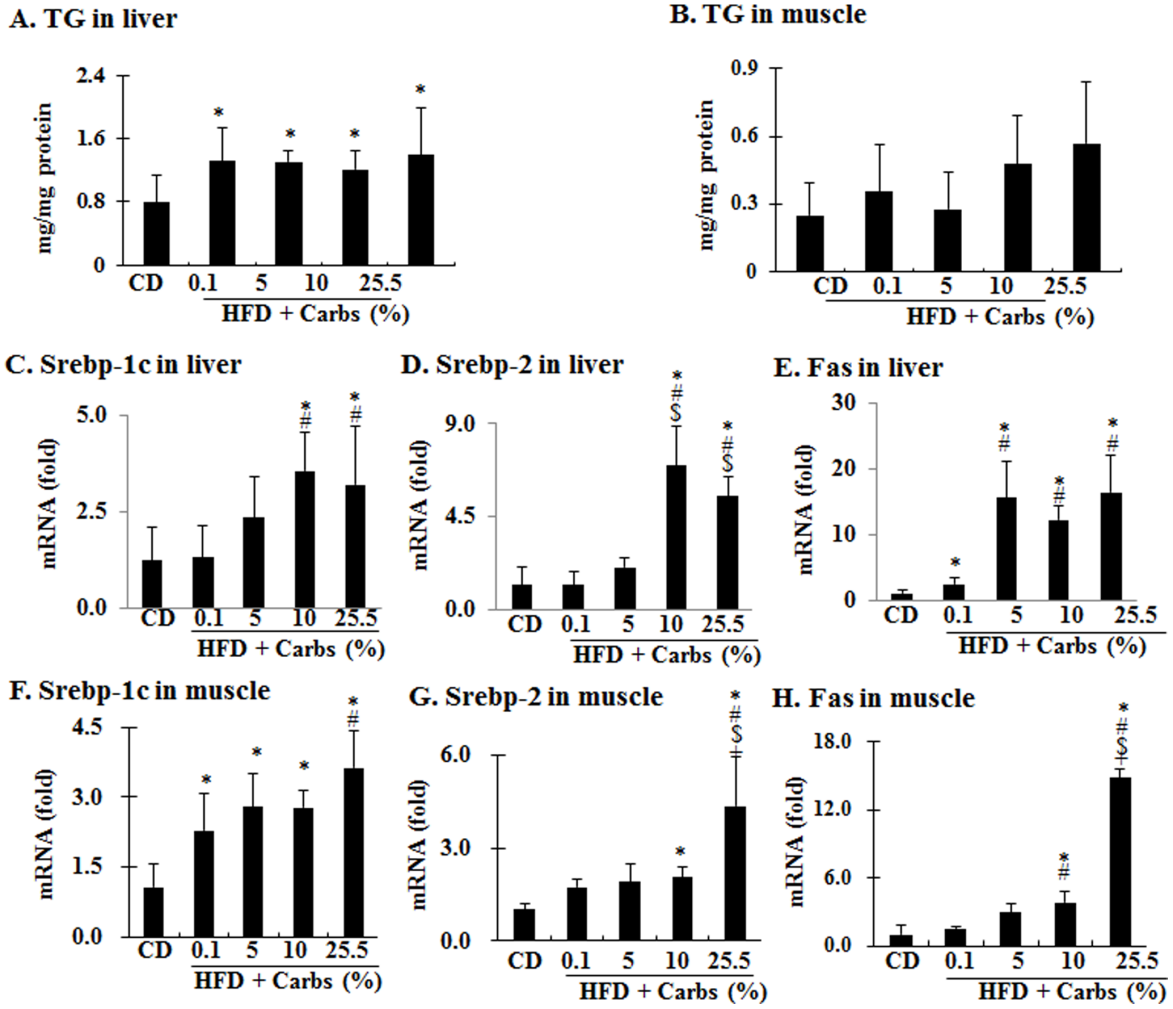
Dietary carb is not necessary for the HFD-induced ectopic fat accumulation in liver and skeletal muscle. At the end of the 5-week experiment, animals were fasted for 8 h and liver and skeletal muscle samples were collected. Content of triglyceride (TG) in liver (**A**) and (**B**) gastrocnemius were quantified. The mRNA levels of key lipogenic genes (Srebp-1c, Srebp-2, and fatty acid synthase (Fas)) in liver (**C–E**) and gastrocnemius (**F–H**) were quantified by using Real Time RT/PCR. Results represent mean ± SD of 4 mice per group. *: P<0.05 vs. CD. #: P<0.05 vs. HFD +0.1% carbs. $: P<0.05 vs. HFD +5% carbs. +: P<0.05 vs. HFD +10% carbs.

We and many others have previously shown that HFD induces ectopic fat accumulation [13],[14], but the contribution of dietary carbohydrates in this process remains unestablished. To address this issue, ectopic accumulation of triglyceride (TG) in liver and gastrocnemius was examined. As shown in **Fig. 5A–B**, TG content was increased in liver and tended to increase in gastrocnemius of all HFD groups, but the increasing trend in gastrocnemius did not reach statistical significance. Expression of fatty acid synthase (Fas) in liver was increased even in mice on HFD with little carbohydrates and addition of carbohydrate (5–25.5%) boosted the increase although expressions of SREBP-1c and SREBP-2 increased only in mice on HFD with a high level of dietary carbohydrates (10% and 25.5%) (**Fig. 5C–E**). In skeletal muscle gastrocnemius, expression of Srebp-1c was increased in all groups of HFD similarly with or without carbohydrates while expressions of Srebp-2 and Fas were significantly increased only in mice on HFD with 10% or 26% carbs (**Fig. 5F–H**). Together these results show that HFD stimulates fat accumulation in liver and addition of dietary carbohydrates does not influence the level of HFD-induced ectopic fat accumulation although dietary carbohydrates seem to influence the lipogenic program in both liver and skeletal muscle.

### Dietary Carbs Are Not Necessary For Hfd To Induce Oxidative Stress

**Figure 6:**
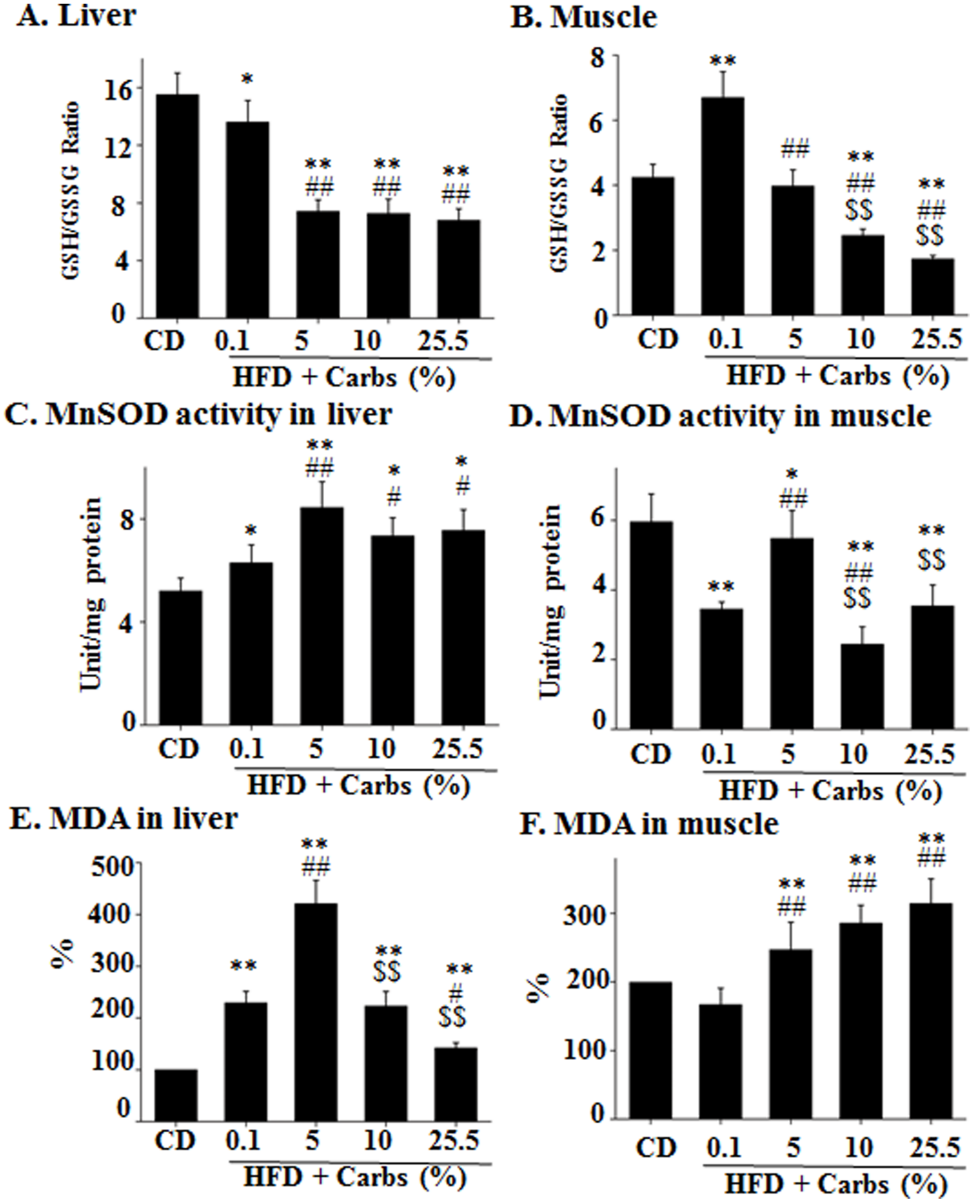
Dietary carb is not necessary for the HFD-induced oxidative stress. Levels of GSH/GSSG ratio, MnSOD activity, malondialdehyde (MDA) in liver (**A, C, E**) and gastrocnemius (**B, D, F**) of the mice described in Fig. 1 were measured and normalized to protein levels of the same samples as detailed in “Methods and Materials”. Results represent results of mean ± SD of 10 animals per group. *: P<0.05 vs. CD. **: P<0.01 vs. CD. #: P<0.05 vs. HFD +0.1% carbs. ##: P<0.01 vs. HFD +0.1% carbs. $$: P<0.01 vs. HFD +5% carbs.

To further investigate the mechanisms of HFD-induced insulin resistance, oxidative stress level was examined in several different ways. As shown in **Fig. 6A–B**, GSH/GSSG ratio was decreased in liver by HFD with little carb and the decrease was much more dramatic when 5%, 10%, or 25.5% carbs were present in the HFD although the dose-dependent manner was not shown. In gastrocnemius, the GSH/GSSG ratio was actually elevated when HFD contained little (0.1%) carb, but decreased when the HFD contained 10% 0r 25.5% carbs. The level of MnSOD activity was increased in liver of mice on HFD that contained little carb (0.1%) but more significantly when the HFD contained 5%, 10%, or 25.5% carbs with no dose dependence (**Fig. 6C**). The activity of MnSOD was reduced in gastrocnemius of mice on HFD with 0.1%, 10%, or 25.5% carbs (**Fig. 6D**). The level of oxidized lipids (MDA) was elevated in liver of mice on all HFD groups without a carbohydrate dose-dependency (**Fig. 6E**). The level of MDA did not increase in gastrocnemius of mice on HFD that contained little (0.1%) carb, but did increase when HFD contained 5%, 10%, or 25.5% carbs (**Fig. 6F**). Together, these results show that HFD with little carb for 5 weeks caused oxidative stress in liver but not in skeletal muscle, but the HFD with 5% or more carbs caused oxidative stress in both liver and skeletal muscle.

### Due To Presence Of Hepatic Gluconeogenesis, Dietary Carb Was Not Necessary For Hfd Induction Of Insulin Resistance

**Figure 7:**
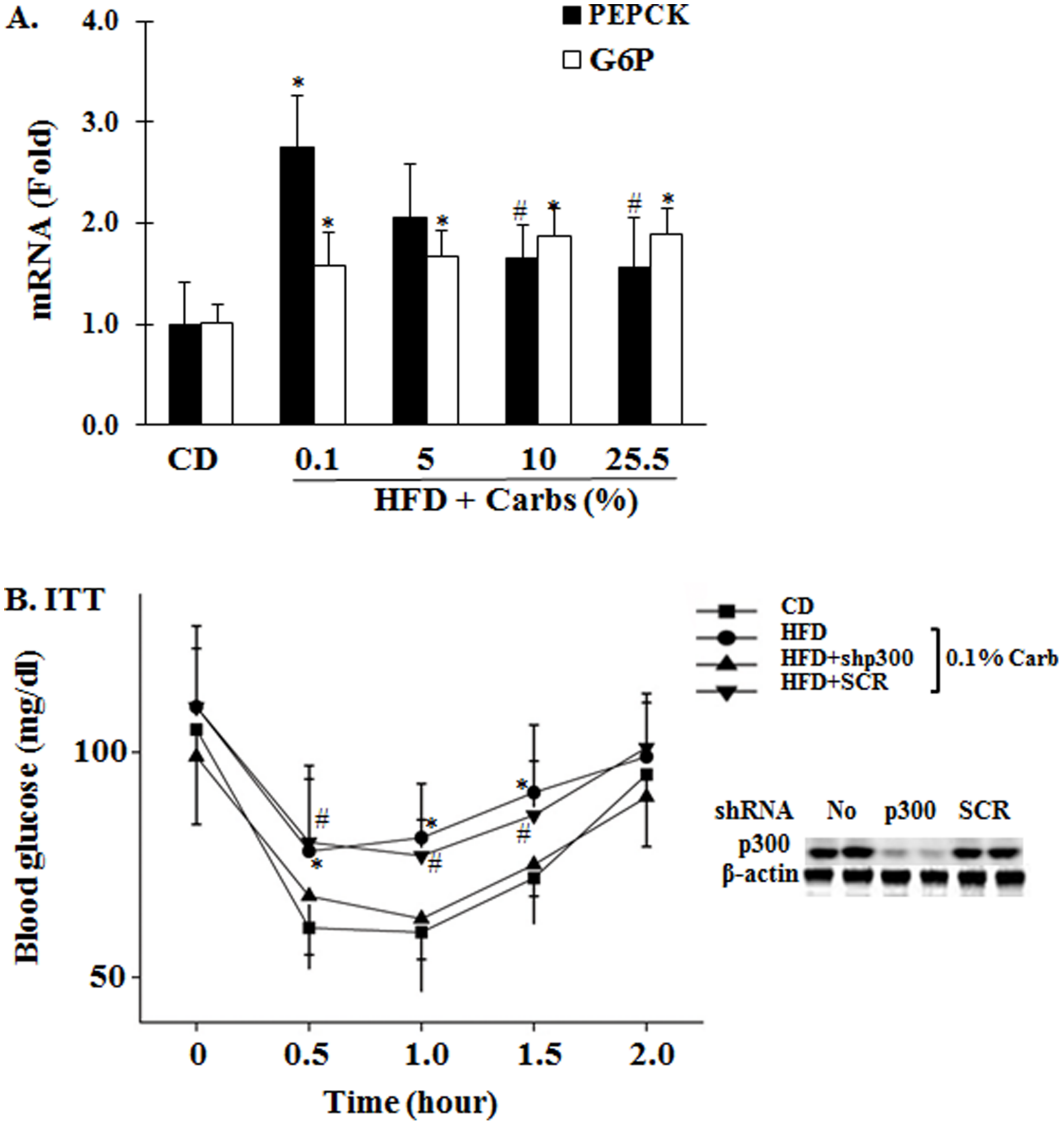
Inhibition of hepatic gluconeogenesis prevented the HFD-induced insulin resistance. (**A**) Levels of key hepatic gluconeogenic genes (PEPCK and glucose-6-phosphatase (G6P) were measured by using RT-PCR as detailed in “Methods and Materials”. Results represent mean ± SD of 4 mice per group. *: P<0.05 vs. CD. #: P<0.05 vs. HFD +0.1% carbs. (**B**) B6 mice were fed either the regular chow diet (CD) *ad libitum* or HFD plus 0.1% carbs for 5 weeks. Some mice were treated with adenoviral shRNA against p300 (10^9^ pfu/mouse) or the control shRNA at the beginning of week 5. After an 8 h fast, IIT was performed. Protein levels of p300 in liver were verified by using immunoblotting. Results represent mean ± SD of 5 mice per group. *: P<0.05 vs. CD. #: P<0.05 vs. HFD + shRNAp300 or CD.

To further investigate the mechanisms of insulin resistance induced by HFD, we examined expression of key gluconeogenic genes. As shown in **Fig. 7A**, expressions of both PEPCK and G-6-Pase were increased by HFD, and the increase in PEPCK was most significant in animals on HFD that contained little (0.1%) carb. To determine the contribution of gluconeogenesis to the HFD-induced insulin resistance, hepatic gluconeogenesis was reduced by knocking down the p300 gene as previously described [22]. As shown in **Fig. 7B**, HFD with little dietary carb induced insulin resistance, and this induction was totally prevented by administration of adenoviral shRNA against p300 but not by the control shRNA. These results demonstrate that dietary carb is not necessary for HFD induction of insulin resistance due to presence of hepatic gluconeogenesis.

## Discussion

In investigating the effect of dietary composition on the development of insulin resistance in mice in this study, we made several novel findings.

First, dietary carb is not required for the HFD-induced weight gain. It is known that B6 mice on HFD intake more calories than the control mice on chow diet and leads to increased body weight gain [11],[12]. In addition to the excess ingestion of calories, decreased thermogenesis is another contributor to the increased body weight gain [32]. Furthermore, it has been previously shown that HFD can promote weight gain by increasing feed efficiency [30]. In this study, mice on HFD with any amount of carb (0.1%–25.5%) ingested fewer calories compared to the controls on chow diet (**Fig. 1A**), but mice on HFD of all groups gained more body weight than controls relatively or absolutely; and the gain is proportional to the amount of carbs in the diet (**Fig. 1C**). Since the lipogenic program, fat accumulation, and the ratio of white fat/body weight (**Fig. 1D** and **5**) were all increased in animals on HFD of all groups, the increased lipogenesis is likely the main contributor to the increased body weight gain in mice of HFD. Besides, the thermogenesis program was decreased because expression of both UCP1 and PGC-1α was reduced in brown fat of mice on HFD of all groups (data not shown). The inhibition of thermogenic program was likely due to hyperinsulinemia in these animals because it has previously been shown that inhibition of insulin secretion with diazoxide prevented the HFD-induced decrease in the expression of β3-adrenergic receptor, which is a potent stimulator of PGC-1α and UCP1 expression in brown fat [33]. Similarly, HFD decreased expression of UCP 2 and UCP3 in skeletal muscle (data not shown). Thus, animals do not need to take more calories to gain more body weight when they are on HFD.

Second, dietary carb is not required for the HFD-induced insulin resistance because of the presence of hepatic glucose production via gluconeogenesis. Many studies have shown that high-carb diets are associated with insulin resistance and T2DM [17]. It has been well conceived that dietary carbs can increase insulin secretion, which subsequently stimulate lipogenesis and then cause insulin resistance [17],[18]. However, it is currently unestablished whether or not dietary carb is necessary for the diet-induced insulin resistance. Results from this study show that mice on HFD containing little carb (0.1%) developed severe insulin resistance (**Fig. 2**). How did that happen? To replace the carb in HFD, ∼42% calories of the HFD used in this study was from proteins. Protein-rich diets have been shown to induce insulin resistance probably through activation of mTOR and S6K signaling pathway [34],[35]. However, activity of mTOR in animals of this study was not altered (**Fig. 3**). Therefore, the mTOR-S6K signaling pathway was not the likely mechanism of insulin resistance of this study. It is noteworthy that hepatic gluconeogenesis program was significantly increased in mice on HFD with little carbs and inhibition of hepatic gluconeogenesis by knocking down p300 completely prevented the HFD-induced insulin resistance (**Fig. 7**). In other words, glucose from gluconeogenesis is sufficient to stimulate sufficient secretion of insulin, which is necessary for the fat- or glucose-induced insulin resistance [14],[24].

Third, a very small amount of dietary carb is sufficient to promote the HFD-induced insulin resistance to a maximal level. Although it is known that dietary carbs play a key role in the development of insulin resistance and T2DM, it is unknown how much dietary carb is too much or sufficient to enhance insulin resistance in humans or animals on HFD. Our results show that a very small amount of dietary carb (5%) in HFD can worsen insulin resistance dramatically, and 10% carb in HFD can cause the maximal level of insulin resistance (**Fig. 2**). With the increase of carbohydrates in the diet there was a decrease in the level of dietary proteins, it was possible although not likely that the increased insulin resistance in mice on HFD with higher levels of carbohydrates might be caused by the decreased protein level in the HFD. However, considering the unaltered mTOR activation level (**Fig. 3B**) and prevention of the HFD-induced insulin resistance by reducing gluconeogenesis (**Fig. 7**), we believe that the effect of changes in protein level is not likely the primary contributor to the changes in insulin sensitivity in this study. In any way, our results may help explain why all kinds of diets are out there with conflicting results. Some studies show low carb diets protects against insulin resistance and T2DM while others show the opposite [17],[20],[21]. That is likely due to the different amounts of carb in the so-called low-carb diets. The amount of carbs in some of those assumed low carb might contain enough carbs to stimulate insulin secretion and induce insulin resistance while that of other low-carb diets is not sufficient to enhance insulin secretion and induce insulin resistance.

Fourth, the lipogenic program (SRBEPs and Fas) was stimulated in all HFD groups with or without dietary carbs and was enhanced by dietary carbs in a dose-dependent manner. The lipogenic program is predominantly stimulated by insulin through the central regulator SREBP-1c and its down-stream effector Fas [36]. Therefore, it is easy to appreciate the dose-dependent increase of lipogenic program by dietary carbs in HFD. However, the increased lipogenic program and ectopic fat accumulation of animals on HFD with little carb seems puzzling. But considering the elevated gluconeogenic program and increased fasting insulin level in those mice, it should be understandable. Typically, HFD-induced ectopic fat accumulation occurs in both liver and skeletal muscle as previously shown [13],[14]. In this study, fat accumulation in liver was obvious but did not reach a statistical significance in skeletal muscle for an unclear reason. Mitochondria-derived oxidative stress plays a critical role in the development of insulin resistance induced by fat or cytokines [37]–[40]. We and others have previously shown that chronic exposure to excess insulin can induce mitochondria-derived oxidative stress through long chain-acyl CoAs or/and cholesterol [14],[41]. Since animals on HFD with or without dietary carbs all had hyperinsulinemia (**Fig. 2**) and hyperlipidemia (data not shown), it is evident that they would all have oxidative stress (**Fig. 6**).

In summary, our results show that dietary carb is not necessary for the HFD-induced insulin resistance because of the presence of hepatic gluconeogenesis but a very small amount of it (5%) can dramatically worsen the HFD-induced insulin resistance and 10% carb in the HFD can induce a maximal level of insulin resistance. Fat is essential for development of insulin resistance.
